# Secondary Resonance Energy Harvesting with Quadratic Nonlinearity

**DOI:** 10.3390/ma13153389

**Published:** 2020-07-31

**Authors:** Guoce Zhang, Bo Zhang

**Affiliations:** Department of Civil Engineering, Hainan University, Haikou 570228, China; bzhang7@hotmail.com

**Keywords:** Galerkin method, piezoelectric energy harvester, nonlinear boundary condition, secondary resonance, quadratic nonlinearity

## Abstract

Piezoelectric energy harvesters can transform the mechanical strain into electrical energy. The microelectromechanical transformation device is often composed of piezoelectric cantilevers and has been largely experimented. Most resonances have been developed to harvest nonlinear vibratory energy except for combination resonances. This paper is to analyze several secondary resonances of a cantilever-type piezoelectric energy harvester with a tip magnet. The conventional Galerkin method is improved to truncate the continuous model, an integro-partial differential equation with time-dependent boundary conditions. Then, more resonances on higher-order vibration modes can be obtained. The stable steady-state response is formulated approximately but analytically for the first two subharmonic and combination resonances. The instability boundaries are discussed for these secondary resonances from quadratic nonlinearity. A small damping and a large excitation readily result in an unstable response, including the period-doubling and quasiperiodic motions that can be employed to enhance the voltage output around a wider band of working frequency. Runge–Kutta method is employed to numerically compute the time history for stable and unstable motions. The stable steady-state responses from two different methods agree well with each other. The outcome enriches structural dynamic theory on nonlinear vibration.

## 1. Introduction

Applying pressure to piezoelectric materials causes them to produce an electric charge. The piezoelectric material would contract or expand in an electric field. Since their discovery, the principles of the piezoelectric effect have caught many scientists’ attention [1,2]. Owing to the piezoelectricity, piezoelectric energy harvesters are invented to transform mechanical vibration into electrical energy. This kind of electromechanical device can provide a potential solution to capture mechanical energy for operating self-sustained low-power electronics. The technology has not only monetary gains in the reduction of the maintenance costs by replacing batteries, but also ecological ramifications in the reduction of the chemical waste [3].

However, the voltages generated by the piezoelectricity and its inverse are usually small [4,5]. More voltage output is desirable in piezoelectric instruments, including ultrasonic medical transducers, ultrasonic cleaning components, ultrasonic welding components, and accelerometers. To this end, cantilevered piezoelectric energy harvesters have been experimentally investigated in a wide range [6]. Linear energy harvesters efficiently work merely at the natural frequency. The piezoelectric cantilever can be designed to match the resonant frequency for a large response amplitude [7]. Such a design has a single peak around a narrow band of frequency. The amount of generated voltage is quite low when the energy harvester works off resonance. The large voltage output around a wide band of operating frequency has been of great concern [8].

Compared with linear energy harvesters, nonlinear devices are promising to extend the working frequency to a wider range [9,10]. Magnetic nonlinearity is often employed to improve cantilevered piezoelectric energy harvester [11,12]. This field encompasses mechanics, material science, and electrical circuitry, and has captivated both academics and industrialists. Over the past two decades, primary-harmonic [13,14], superharmonic [15,16], and subharmonic resonances [17] have been discussed analytically, numerically, or experimentally. Internal resonances are also involved in enhancing the performance of harvesting mechanical energy [18,19]. To the authors’ knowledge, the combination resonances have not been developed to capture vibratory energy [20]. The efforts have been made on them as well as two subharmonic resonances for an electromechanical system with quadratic nonlinearity in this article.

It has not only experimental value but also theoretical significance to investigate a cantilever-type piezoelectric energy harvester with tip magnets [21,22]. For a beam-type structure, the mathematical model is a partial differential equation [23,24]. At the free end of a slender cantilever, the magnetic force from external magnets serves nonlinear boundary conditions [25,26]. If the cantilevered structure was excited, the boundary condition is explicitly relevant to time. An inertial force lasts as long as the accelerating tip magnets do. The entire beam element could be modeled as a fourth-order partial differential equation with a time-dependent boundary condition [27,28]. The complex boundary condition makes the nonlinear dynamic problem difficult to solve. However, the stable steady-state responses are pursued analytically for the first two subharmonic resonances and combination resonances with quadratic nonlinearity.

As a matter of fact, the conventional Galerkin method is under consideration, as an exact solution to the dynamic problem subjected to initial conditions does not yet exist [29]. Unfortunately, the Galerkin method is constrained by the time-dependent boundary condition [25,26,27,28]. Even though, in a family of trial functions, each spatial function satisfies the nonlinear boundary condition, a combination of these trial functions will generally not do so. To circumvent this problem, weight functions are used prior to trial functions [30]. Combined with the nonlinear boundary condition, modal functions can be chosen as weight functions to obtain a weighted average of the governing equation. It is followed by expressing a solution into the form of a weighted sum of trial functions. The improved Galerkin method is applied to analyze the above-mentioned secondary resonances.

Under these resonances, damping plays an important role in nonlinear vibration [31]. When damping parameters are designed well, the response is unstable for a periodic external excitation. In particular, the vibration can grow without bound on the subharmonic or combination resonance from quadratic nonlinearity. These secondary resonances are potentially useful to improve electrical output. This is just our motivation to harvest unstable mechanical energy based on secondary resonances. To reveal the effect of material damping on them, the Kelvin–Voigt damping model is applied in the cantilevered piezoelectric energy harvester [28]. The stability is analyzed by the method of multiple scales. The Runge–Kutta method is used to quantitatively examine the stable steady-state response.

To sum up, the task of our work is to develop secondary resonances to harvest more mechanical energy around a wider range of working frequencies. The effects of damping and excitation on secondary resonances from quadratic nonlinearity are discussed in detail. Their stability theories are analytically proposed to better design the physical parameters of the piezoelectric cantilever. These discussions are on the theoretical basis of mathematical analyses, structural dynamics, and mechanics of materials. All the formulas are derived from the original mathematical model by hand. All the numerical simulations are carried out by the C programming language. The core findings are as follows:

(1) A novel methodology is proposed to deal with the issue of continuum mechanics with time-dependent boundary conditions. The mathematical model is an integro-partial differential equation. The conventional orthogonal relationships between any two modal functions are invalid.

(2) The summed combination resonance is first developed to harvest mechanical energy while the differential combination resonance does not exist. These outcomes cannot be derived from the traditional single-degree-of-freedom system used constantly in the field of energy harvesting.

(3) The subharmonic resonance is another way to improve the bandwidth of working frequency. The in-depth analyses can better explain those results observed in experiments, and approximately forecast the working conditions for energy harvesters based on stability theories.

(4) Some interesting phenomena, such as the period-doubling and the quasiperiodic motions, are numerically predicted in the hope of more experimental computations. The hard piezo-materials with higher mechanical quality factors might be beneficial for experimental verification and real use.

The rest is structured as follows. The nonlinear model of a cantilevered piezoelectric energy harvester subjected to a tip magnetic force is established in Section 2. Its natural frequencies and modal functions are calculated and used in the application of the improved Galerkin method. Section 3 analyzes the combination resonances based on a truncation system. The method of two times scales is employed to deal with the governing equation and its stability condition. Two subharmonic resonances are analyzed in Section 4. Section 5 explains a previous experiment by others, and discusses the effects of damping coefficient, excitation amplitude, and maximal voltage on secondary resonance energy harvesting with quadratic nonlinearity. Section 6 ends this paper with some concluding remarks.

## 2. Governing Equations

### 2.1. The Equation of Absolute Motion

**Figure 1:**
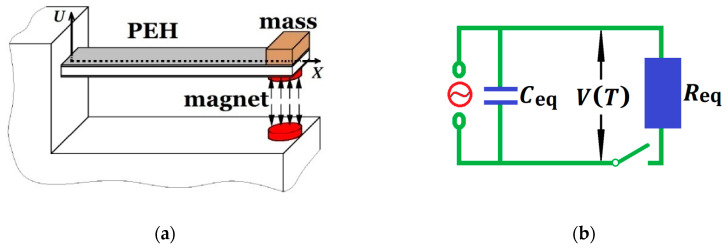
The electromechanical system for piezoelectric energy harvesting (PEH): (**a**) cantilevered structure; (**b**) electric circuit.

The considered electromechanical device is primarily composed of a cantilevered structure (Figure 1a) with a general electric circuit (Figure 1b). The slender and uniform cantilever, clamped at a framework, is assumed to be of a rectangular cross-section. A tip-proof mass is attached to adjust the natural frequency for real use. A cylindrical permanent magnet is fixed at the right end of the beam-type structure. The magnet, modeled as a portion of the tip lumped mass, is perpendicularly repelled by a similar cylindrical magnet which is attached to the framework. The structural parameters are the same compared to [3], as described in Table 1.

It is assumed that the two applied cylindrical magnets are both with height *H* and radius *R*. The initial distance between them is symbolically denoted by *D* while the entire structure is just equipped. *ρ* is the residual magnetic flux density. *S* is the common area between the two magnets. *μ* is the permeability of air. The magnetic force between the two cylindrical magnets was formulated in the form of [3,7,32]

(1) $$F_{m}\left(D\right)=\frac{\rho_{}^{2}S_{}^{2}{\left(R+H\right)}^{2}}{\pi \mu H_{}^{2}}\left[\frac{1}{D^{2}}+\frac{1}{{\left(D+2H\right)}^{2}}-\frac{2}{{\left(D+H\right)}^{2}}\right].$$

In addition, an extremely thin piezoelectric ceramic patch covers the whole surface of the cantilever. Two electrodes cover the opposite transversal faces of the piezoceramic (PZT) layer. The two electrodes will be connected to a resistive load. When the piezoelectric device is subjected to mechanical stress, an electric charge is generated. As illustrated in Figure 1b, the circuitry includes a resistive load as well as an internal capacitance because the electrode pair brackets the PZT layer. According to Kirchhoff laws, the circuit equation can be expressed as [25,26]

(2) $$C_{\mathrm{eq}}\frac{dV\left(T\right)}{dT}+\frac{V\left(T\right)}{R_{\mathrm{eq}}}=\Theta \int_{0}^{L}\frac{\partial_{}^{3}U\left(X,T\right)}{\partial X_{}^{2}\partial T}dX,$$

where 0 < *X* < *L*, the piezoelectric energy harvester offers the current source, *C*_eq_ represents the equivalent capacitance, *R*_eq_ represents the equivalent resistance, Θ represents the electromechanical coupling coefficient, *V*(*T*) represents the voltage output across the resistive load at the time of *T*, *X* is the spatial coordinate and *U*(*X*, *T*) represents the transversal displacement from the horizontal axis where the clamped end of the cantilever is the original point, as depicted in Figure 1.

The Kelvin–Voigt damping model is applied to contribute to the material damping effect of the system of viscosity coefficient Λ. A tip proof of mass *m*_t_ (together with the tip magnet) is attached to the cantilever of mass *m*_b_, length *L*, Young’s modulus *E*, and area moment of inertial *I*. The internal bending moment of the cantilever can be represented as [25,26]

(3) $$M\left(X,T\right)=EI\frac{\partial_{}^{2}U\left(X,T\right)}{\partial X_{}^{2}}+\Lambda I\frac{\partial_{}^{3}U\left(X,T\right)}{\partial X_{}^{2}\partial T}-\Theta V\left(T\right)\left[H\left(X-L\right)-H\left(X\right)\right],$$

where *H*( ) is the Heaviside step function, which is defined as the integral of the Dirac delta function.

If 0 < *X* < *L*, the equation of absolute motion is

(4) $$\frac{m_{b}}{L}\left[g+\frac{\partial_{}^{2}U\left(X,T\right)}{\partial T_{}^{2}}\right]+EI\frac{\partial_{}^{4}U\left(X,T\right)}{\partial X_{}^{4}}+\Lambda I\frac{\partial_{}^{5}U\left(X,T\right)}{\partial X_{}^{4}\partial T}=\Theta V\left(T\right)\left[\frac{d\delta \left(X-L\right)}{dX}-\frac{d\delta \left(X\right)}{dX}\right]\;,$$

where g is the acceleration of gravity, and the Dirac delta function satisfies

(5) $$\delta \left(X\right)=\{\begin{array}{cc} 0 & X\neq 0\\ +\infty & X=0 \end{array};\;\int_{-\infty }^{+\infty }\delta \left(X\right)dX=1\;.$$

The following is the boundary conditions at *X* = 0 or *L*. For a slender cantilever, the small tip magnet can be viewed as a point mass, and the magnetic force serves the nonlinear boundary condition at the right end of the cantilever. At its left end, the transversal displacement of the base is assumed to be *B*sin(*ΩT*). Then, the boundary conditions are [25,26,27,28]

(6) $$U\left(X,T\right)=B\sin\left(\Omega T\right);\;\frac{\partial U\left(X,T\right)}{\partial X}=0,$$

at *X* = 0, and

(7) $$\begin{array}{l} EI\frac{\partial_{}^{2}U\left(X,T\right)}{\partial X_{}^{2}}=0;\;\\ m_{t}\frac{\partial_{}^{2}U\left(X,T\right)}{\partial T_{}^{2}}=EI\frac{\partial_{}^{3}U\left(X,T\right)}{\partial X_{}^{3}}+I\Lambda \frac{\partial_{}^{4}U\left(X,T\right)}{\partial X_{}^{3}\partial T}-m_{t}g+F_{m}\left(D-B\sin\left(\Omega T\right)+U\left(X,T\right)\right), \end{array}$$

at *X* = *L*. Equation (7) offers the nonlinearity, which is explicitly dependent on time.

Introduce dimensionless parameters as follows.

(8) $$\begin{array}{c} x=\frac{X}{L},\;t=\frac{T}{L}\sqrt{\frac{EI}{Lm_{b}}},\;u=\frac{U}{L}+\frac{m_{b}gX^{2}\left(X^{2}-4LX+6L^{2}\right)+4m_{t}gLX^{2}\left(3L-X\right)}{24EIL^{2}},\\ \beta =\frac{B}{L},\;\omega =\Omega \sqrt{\frac{m_{b}L^{3}}{EI}},\;c_{0}=\frac{\rho^{2}S^{2}{\left(H+R\right)}^{2}}{\pi \mu EIH^{2}},\;h=\frac{H}{L},\;d=\frac{D}{L}-\left(\frac{m_{b}}{8}+\frac{m_{t}}{3}\right)\frac{gL^{2}}{EI},\;\\ \alpha =\frac{\Lambda }{L}\sqrt{\frac{I}{ELm_{b}}},\;\eta =\frac{m_{t}}{m_{b}},\;v=\frac{V}{e},\;c=\frac{e^{2}L^{2}C_{\mathrm{eq}}}{EI},\;\kappa =\frac{e^{2}L^{3}}{EIR_{\mathrm{eq}}}\sqrt{\frac{Lm_{b}}{EI}},\;\theta =\frac{e\Theta L^{2}}{EI},\; \end{array}$$

where *e* is the unit voltage (one volt). The dimensionless value *η* represents the ratio of the tip mass to the mass of the cantilever.

From Equations (2), (4), and (8), the application of the Euler–Bernoulli beam theory yields the dimensionless governing equation, which is represented algebraically as

(9) $$\ddot{u}+u^{\prime \prime }{}^{\prime \prime }+\alpha {\dot{u}}^{\prime \prime }{}^{\prime \prime }=\theta v\left[\delta^{\prime }\left(x-1\right)-\delta^{\prime }\left(x\right)\right];\;c\dot{v}+\kappa v=\theta \int_{0}^{1}{\dot{u}}^{\prime \prime }dx\;,$$

where 0 < *x* < 1, the prime denotes the (partial) derivative with respect to *x*, and the overdot denotes the (partial) derivative with respect to *t*.

The corresponding boundary conditions are simplified as

(10) $$u\left(0,t\right)=\beta \sin\left(\omega t\right);\;u^{\prime }\left(0,t\right)=\;u^{\prime \prime }\left(1,t\right)=0;\;\eta \ddot{u}\left(1,t\right)=u^{\prime \prime \prime }\left(1,t\right)+\alpha {\dot{u}}^{\prime \prime \prime }\left(1,t\right)+F\left(d-\beta \sin\left(\omega t\right)+u\left(1,t\right)\right),$$

where the dimensionless magnetic force function is

(11) $$F\left(d\right)=c_{0}\left[d^{-2}+{\left(d+2h\right)}^{-2}-2{\left(d+h\right)}^{-2}\right].$$

### 2.2. The Equation of Relative Motion

The standard governing equation of relative motion round its equilibrium will be established. The equilibrium position can be analytically derived from Equation (9). To this end, introduce a new coordinate transformation as follows [27].

(12) $$z\left(x,t\right)=u\left(x,t\right)-\beta \sin\left(\omega t\right)\;-A_{e}\left(1.5x^{2}-0.5x^{3}\right),$$

where *A*_e_ satisfies

(13) $$F\left(d+A_{e}\right)=3A_{e}\;\left(\left|A_{e}\right|\leq d\right).$$

Substituting Equation (12) into Equation (9) yields the standard governing equation.

(14) $$\ddot{z}+z^{\prime \prime }{}^{\prime \prime }+\alpha {\dot{z}}^{\prime \prime }{}^{\prime \prime }=\theta v\left[\delta^{\prime }\left(x-1\right)-\delta^{\prime }\left(x\right)\right]+\beta \omega^{2}\sin\left(\omega t\right);\;c\dot{v}+\kappa v=\theta \int_{0}^{1}{\dot{z}}^{\prime \prime }dx.$$

Substituting Equation (12) into Equation (10) yields new boundary conditions.

(15) $$\begin{array}{c} z\left(0,t\right)=z^{\prime }\left(0,t\right)=z^{\prime \prime }\left(1,t\right)=0;\\ z^{\prime \prime \prime }\left(1,t\right)+\alpha {\dot{z}}^{\prime \prime \prime }\left(1,t\right)=\eta \ddot{z}\left(1,t\right)-\eta \beta \omega^{2}\sin\left(\omega t\right)+F\left(d+A_{e}\right)-F\left(d+A_{e}+z\left(1,t\right)\right). \end{array}$$

The Taylor series expansion method is valid to deal with the nonlinear terms in the boundary condition (15).

(16) $$F\left(d+A_{e}\right)-F\left(d+A_{e}+z\left(1,t\right)\right)=\sum_{j=1}^{+\infty }\left[c_{j}z^{j}\left(1,t\right)\right],$$

where *j* = 1, 2, 3, …

(17) $$c_{j}=c_{0}\left(j+1\right){\left(-1\right)}^{j+1}\left[{\left(d+A_{e}\right)}^{-2-j}+{\left(d+A_{e}+2h\right)}^{-2-j}-2{\left(d+A_{e}+h\right)}^{-2-j}\right].$$

### 2.3. Natural Frequencies and Modal Functions

In general, the Galerkin method involves the modal functions of the linearized equation. In the following, the natural frequencies and modal functions are both to be determined. Some of their properties are to be shown. To this end, the standard governing equation goes into a linear form that is independent of the viscoelastic damping, electrical coupling, and external excitation.

(18) $$\ddot{z}+z^{\prime \prime }{}^{\prime \prime }=0;\;z\left(0,t\right)=z^{\prime }\left(0,t\right)=z^{\prime \prime }\left(1,t\right)=0;\;z^{\prime \prime \prime }\left(1,t\right)=\eta \ddot{z}\left(1,t\right)+c_{1}z\left(1,t\right).$$

This linear partial differential Equation (18) has been solved [27]. It can be expressed as

(19) $$z\left(x,t\right)=\sum_{n=1}^{+\infty }\left[\;\phi_{n}\left(x\right)q_{n}\left(t\right)\right],$$

where *n* = 1, 2, 3, … and the modal functions satisfy the following equation.

(20) $$\phi_{n}^{\prime \prime }{}^{\prime \prime }\;\left(x\right)=\omega_{n}^{2}\phi_{n}\left(x\right);\;\phi_{n}\left(0\right)={\phi^{\prime }}_{n}\left(0\right)={\phi^{\prime \prime }}_{n}\left(1\right)=0;\;\phi_{n}^{\prime \prime \prime }\left(1\right)=\left(c_{1}-\eta \omega_{n}^{2}\right)\phi_{n}\left(1\right).$$

where the natural frequencies are obtained from

(21) $$\sqrt{\omega_{n}^{3}}\;\left(1+\cos\sqrt{\omega_{n}}\mathrm{ch}\sqrt{\omega_{n}}\right)=\left(c_{1}-\eta \omega_{n}^{2}\right)\left(\cos\sqrt{\omega_{n}}\mathrm{sh}\sqrt{\omega_{n}}-\sin\sqrt{\omega_{n}}\mathrm{ch}\sqrt{\omega_{n}}\right).$$

For convenience, the *n*th modal function with condition $\phi_{n}\left(1\right)=1$ is in the form of

(22) $$\phi_{n}\left(x\right)=\frac{\left(\sin\sqrt{\omega_{n}}+\mathrm{sh}\sqrt{\omega_{n}}\right)\left[\cos\left(x\sqrt{\omega_{n}}\right)-\mathrm{ch}\left(x\sqrt{\omega_{n}}\right)\right]+\left(\cos\sqrt{\omega_{n}}+\mathrm{ch}\sqrt{\omega_{n}}\right)\left[\mathrm{sh}\left(x\sqrt{\omega_{n}}\right)-\sin\left(x\sqrt{\omega_{n}}\right)\right]}{2\left(\cos\sqrt{\omega_{n}}\mathrm{sh}\sqrt{\omega_{n}}-\sin\sqrt{\omega_{n}}\mathrm{ch}\sqrt{\omega_{n}}\right)}.$$

Here, the integral of the product of two different modal functions satisfies

(23) $$\int_{0}^{1}\phi_{n}\phi_{k}dx=-\eta \left(\omega_{n}^{}\neq \omega_{k}^{}\right).$$

Besides, two other integrals are following.

(24) $$\int_{0}^{1}\phi_{k}z^{\prime \prime }{}^{\prime \prime }dx={\phi_{k}z^{\prime \prime \prime }\;|}_{0}^{1}-{\phi_{k}^{\prime }z^{\prime \prime }|}_{0}^{1}+{\phi_{k}^{\prime \prime }z^{\prime }|}_{0}^{1}-{z\phi_{k}^{\prime \prime \prime }\;|}_{0}^{1}+\int_{0}^{1}z\phi_{k}^{\prime \prime }{}^{\prime \prime }dx=z^{\prime \prime \prime }\left(1,t\right)+\left(\eta \;\omega_{k}^{2}-c_{1}\right)z\left(1,t\right)+\omega_{k}^{2}\int_{0}^{1}z\phi_{k}dx,$$

and

(25) $$\int_{0}^{1}\phi_{k}{\dot{z}}^{\prime \prime }{}^{\prime \prime }dx={\dot{z}}^{\prime \prime \prime }\left(1,t\right)+\left(\eta \;\omega_{k}^{2}-c_{1}\right)\dot{z}\left(1,t\right)+\omega_{k}^{2}\int_{0}^{1}\dot{z}\phi_{k}dx.$$

Equations (23)–(25) is useful in the use of the Galerkin method.

### 2.4. The Application of Galerkin Method

By convention, modal functions can be chosen as both weight and trial functions. At first, a list of modal functions works as weight functions. In order to give a weighted average of Equation (14), it is integrated simply with respect to *x* over [0, 1]. Performing the definite integral of the mechanical component over the closed interval [0, 1] leads to

(26) $$\int_{0}^{1}\ddot{z}\phi_{k}dx+\int_{0}^{1}\phi_{k}z^{\prime \prime }{}^{\prime \prime }dx+\alpha \int_{0}^{1}\phi_{k}{\dot{z}}^{\prime \prime }{}^{\prime \prime }dx+v\theta_{k}=\beta \omega^{2}\sin\left(\omega t\right)\int_{0}^{1}\phi_{k}dx,$$

where

(27) $$\theta_{k}=\theta \phi_{k}^{\prime }\left(1\right)\;.$$

Substituting Equations (24) and (25) into Equation (26) gives

(28) $$\begin{array}{l} v\theta_{k}+\int_{0}^{1}\ddot{z}\phi_{k}dx+\alpha \omega_{k}^{2}\int_{0}^{1}\dot{z}\phi_{k}dx+\left(\eta \;\omega_{k}^{2}-c_{1}\right)\alpha \dot{z}\left(1,t\right)+\alpha {\dot{z}}^{\prime \prime \prime }\left(1,t\right)\\ \;+\omega_{k}^{2}\int_{0}^{1}z\phi_{k}dx+\left(\eta \;\omega_{k}^{2}-c_{1}\right)z\left(1,t\right)+z^{\prime \prime \prime }\left(1,t\right)=\beta \omega^{2}\sin\left(\omega t\right)\int_{0}^{1}\phi_{k}dx. \end{array}$$

According to the boundary conditions (15), the substitution of $z^{\prime \prime \prime }\left(1,t\right)+\alpha {\dot{z}}^{\prime \prime \prime }\left(1,t\right)$ in Equation (28) gives

(29) $$\begin{array}{l} \int_{0}^{1}\ddot{z}\phi_{k}dx+\alpha \omega_{k}^{2}\int_{0}^{1}\dot{z}\phi_{k}dx+\left(\eta \;\omega_{k}^{2}-c_{1}\right)\alpha \dot{z}\left(1,t\right)+\omega_{k}^{2}\int_{0}^{1}z\phi_{k}dx+\left(\eta \;\omega_{k}^{2}-c_{1}\right)z\left(1,t\right)\\ \;+\eta \ddot{z}\left(1,t\right)+\sum_{j=1}^{+\infty }\left[c_{j}z^{j}\left(1,t\right)\right]+v\theta_{k}=\left(\eta +\int_{0}^{1}\phi_{k}dx\right)\beta \omega^{2}\sin\left(\omega t\right). \end{array}$$

The following is the second step, choosing appropriate trial functions. As a general rule, it is convenient to assume expansions for the relative displacement in the form of the weighted sum of linear modes (19). To perform calculations, *N*-term truncation that has a finite number of terms is used.

(30) $$z\left(x,t\right)=\sum_{n=1}^{N}\left[\;\phi_{n}\left(x\right)q_{n}\left(t\right)\right].$$

The truncation with the use of Equation (27) makes the electrical component of Equation (14) become

(31) $$c\dot{v}+\kappa v=\theta \sum_{n=1}^{N}\left[{\dot{q}}_{n}\int_{0}^{1}\phi_{n}^{\prime \prime }\left(x\right)dx\right]\;=\sum_{n=1}^{N}\left(\theta_{n}{\dot{q}}_{n}\right).$$

Meanwhile, the mechanical counterpart (29) becomes

(32) $$\left({\ddot{q}}_{k}+\alpha \;\omega_{k}^{2}{\dot{q}}_{k}+\omega_{k}^{2}q_{k}\right)\eta_{2k}\;-\alpha c_{1}\sum_{n=1}^{N}{\dot{q}}_{n}\;+\sum_{j=2}^{\infty }c_{j}{\left(\sum_{n=1}^{N}q_{n}\right)}^{j}+v\theta_{k}=\omega^{2}\beta \eta_{1k}\sin\left(\omega t\right),$$

where *n* = 1,2,3, … *N*, and

(33) $$\eta_{nk}=\eta +\int_{0}^{1}\phi_{k}^{n}\left(x\right)dx.$$

Equations (31) and (32) are the discrete truncation system that is being sought and to be analyzed.

## 3. Combination Resonances

### 3.1. The Multiscale Analyses

To investigate combination resonances, at least two-term truncation is in need. When *N* = 2, the governing equations become

(34) $$\begin{array}{c} c\dot{v}+\kappa v=\theta_{1}{\dot{q}}_{1}+\theta_{2}{\dot{q}}_{2};\\ \left({\ddot{q}}_{1}+\alpha \;\omega_{1}^{2}{\dot{q}}_{1}+\omega_{1}^{2}q_{1}\right)\eta_{21}\;-\alpha c_{1}\left({\dot{q}}_{1}\;+{\dot{q}}_{2}\;\right)+\sum_{j=2}^{\infty }c_{j}{\left(q_{1}+q_{2}\right)}^{j}+v\theta_{1}=\omega^{2}\beta \eta_{11}\sin\left(\omega t\right);\\ \left({\ddot{q}}_{2}+\alpha \;\omega_{2}^{2}{\dot{q}}_{2}+\omega_{2}^{2}q_{2}\right)\eta_{22}\;-\alpha c_{1}\left({\dot{q}}_{1}\;+{\dot{q}}_{2}\;\right)+\sum_{j=2}^{\infty }c_{j}{\left(q_{1}+q_{2}\right)}^{j}+v\theta_{2}=\omega^{2}\beta \eta_{12}\sin\left(\omega t\right). \end{array}$$

As for a weakly nonlinear energy harvester, the solution to Equation (34) is sought in the form of

(35) $$v\left(t\right)=\varepsilon V_{1}\left(T_{0},T_{1}\right)+\varepsilon^{2}V_{2}\left(T_{0},T_{1}\right)+O\left(\varepsilon^{3}\right);\;q_{n}\left(t\right)=\varepsilon Q_{n1}\left(T_{0},T_{1}\right)+\varepsilon^{2}Q_{n2}\left(T_{0},T_{1}\right)+O\left(\varepsilon^{3}\right),$$

where *ε* is a small parameter, the new time variable *T*_0_ is equal to *t*, and *T*_1_ = *εt*, which is a slower time scale than *T*_0_. *T*_1_ characterizes the modulation of the amplitudes and phases due to viscoelastic damping and possible resonances.

The derivatives with respect to time *t* become

(36) $$\frac{d}{dt}=D_{0}^{}+\varepsilon D_{1}^{}+\varepsilon_{}^{2}D_{2}^{}+\;\cdots ;\;\frac{d_{}^{2}}{dt_{}^{2}}=D_{0}^{2}+2\varepsilon D_{0}^{}D_{1}^{}+\varepsilon_{}^{2}\left(D_{1}^{2}+2D_{0}^{}D_{2}^{}\right)+\cdots ,$$

where

(37) $$D_{j}^{}=\frac{\partial }{\partial T_{j}},\;D_{j}^{2}=\frac{\partial_{}^{2}}{\partial T_{j}^{2}},\;\cdots$$

When the frequency of the external excitation is away from the natural frequency, the effect of the excitation is small unless its amplitude is hard. To examine subharmonic and combination resonances, the excitation should appear in the lowest-order perturbation equation. To this end, it is necessary to reorder the electromechanical coupling coefficient, viscoelastic damping, and external excitation as follows.

(38) θ↔εθ;         α↔εα;         β↔εβ.

Substituting Equations (35)–(38) into Equation (34) and equating the coefficient of *ε* on both sides yield

(39a) $$\;cD_{0}^{}V_{1}+\kappa V_{1}=0,$$

(39b) $$\left(D_{0}^{2}Q_{11}+\omega_{1}^{2}Q_{11}\right)\eta_{21}\;=\omega^{2}\beta \eta_{11}\sin\left(\omega t\right);\;\left(D_{0}^{2}Q_{21}+\omega_{2}^{2}Q_{21}\right)\eta_{22}\;=\omega^{2}\beta \eta_{12}\sin\left(\omega t\right)\;.$$

Equating the coefficient of the square of epsilon on both sides can give

(40a) $$cD_{0}^{}\;V_{2}+\kappa V_{2}=\theta_{1}D_{0}^{}Q_{11}+\theta_{2}D_{0}^{}Q_{21}-cD_{1}^{}\;V_{1},$$

(40b) $$\begin{array}{l} \left(D_{0}^{2}Q_{12}+\omega_{1}^{2}Q_{12}\right)\eta_{21}\;=\alpha \left(c_{1}-\omega_{1}^{2}\eta_{21}\;\right)\;D_{0}^{}Q_{11}+\alpha c_{1}D_{0}^{}Q_{21}\;-2\eta_{21}D_{0}^{}D_{1}^{}Q_{11}-c_{2}{\left(Q_{11}+Q_{21}\right)}^{2}-\theta_{1}V_{1};\\ \left(D_{0}^{2}Q_{22}+\omega_{2}^{2}Q_{22}\right)\eta_{22}\;=\alpha \left(c_{1}-\;\omega_{2}^{2}\eta_{22}\right)D_{0}^{}Q_{21}+\alpha c_{1}D_{0}^{}Q_{11}-2\eta_{22}D_{0}^{}D_{1}^{}Q_{21}-c_{2}{\left(Q_{11}+Q_{21}\right)}^{2}-\theta_{2}V_{1}. \end{array}$$

The general solution of Equation (39a) is

(41) $$V_{1}\left(T_{0},T_{1}\right)=\;Y_{0}\left(T_{1}\right)\exp\left(-\frac{\kappa }{c}T_{0}\right)\to 0\;\left(t\to \infty \right),$$

where *Y*_0_(*T*_1_) is a function controlled by initial conditions. For the steady-state motion, the general solution of Equation (39a,b) is taken in the form of

(42) $$Q_{11}=Y_{1}\left(T_{1}\right)e^{i\omega_{1}T_{0}}+\frac{a_{1}^{}}{2i}e^{i\omega T_{0}}+\mathrm{cc};\;Q_{21}=Y_{2}\left(T_{1}\right)e^{i\omega_{2}T_{0}}+\frac{a_{2}^{}}{2i}e^{i\omega T_{0}}+\mathrm{cc};\;V_{1}=0,$$

where cc is the complex conjugate of all the preceding terms, and

(43) $$a_{1}^{}=\frac{\omega^{2}\beta \eta_{11}}{\eta_{21}\;\left(\omega_{1}^{2}-\omega^{2}\right)};\;a_{2}^{}=\frac{\omega^{2}\beta \eta_{12}}{\eta_{22}\;\left(\omega_{2}^{2}-\omega^{2}\right)}.$$

Then, the second-order Equation (40a) becomes

(44) $$c\;D_{0}^{}\;V_{2}+\kappa V_{2}=i\theta_{1}^{}\omega_{1}Y_{1}e^{i\omega_{1}T_{0}}+i\theta_{2}^{}\omega_{2}Y_{2}e^{i\omega_{2}T_{0}}+0.5\omega \left(a_{1}^{}\theta_{1}^{}+a_{2}^{}\theta_{2}^{}\right)e^{i\omega T_{0}}+\mathrm{cc},$$

whose solution is

(45) $$V_{2}=\frac{\omega \left(a_{1}^{}\theta_{1}+a_{2}^{}\theta_{2}\right)}{2\left(\kappa +ic\omega \right)}e^{i\omega T_{0}}+\frac{i\theta_{1}^{}\omega_{1}}{\kappa +ic\omega_{1}}Y_{1}^{}e^{i\omega_{1}T_{0}}+\frac{i\theta_{2}^{}\omega_{2}}{\kappa +ic\omega_{2}}Y_{2}^{}e^{i\omega_{2}T_{0}}+\mathrm{cc}.$$

In the next sections, two subharmonic resonances and combination resonances will be analyzed on the basis of Equation (40b).

### 3.2. The Stability Analyses

In the beginning, the summed harmonic resonance is discussed. Introduce the detuning parameter according to

(46) $$\varepsilon \sigma_{0}=\;\omega -\left(\omega_{1}+\omega_{2}\right).$$

Hence,

(47) $$\left(\omega -\omega_{1}\right)T_{0}=\;\left(\omega_{2}+\varepsilon \sigma_{0}\right)t=\omega_{2}T_{0}+\sigma_{0}T_{1};\;\left(\omega -\omega_{2}\right)T_{0}=\;\left(\omega_{1}+\varepsilon \sigma_{0}\right)t=\omega_{1}T_{0}+\sigma_{0}T_{1}.$$

The second-order Equation (40b) becomes

(48) $$\begin{array}{l} \left(D_{0}^{2}Q_{12}+\omega_{1}^{2}Q_{12}\right)\eta_{21}\;=\;i\left[\alpha \omega_{1}\left(c_{1}-\;\omega_{1}^{2}\eta_{21}\right)Y_{1}-2\omega_{1}\eta_{21}D_{1}^{}Y_{1}+c_{2}\left(a_{1}^{}+a_{2}^{}\right){\bar{Y}}_{2}e^{i\sigma_{0}^{}T_{1}}\right]e^{i\omega_{1}T_{0}}+\mathrm{cc}+NST;\\ \left(D_{0}^{2}Q_{22}+\omega_{2}^{2}Q_{22}\right)\eta_{22}\;=i\left[\alpha \omega_{2}\left(c_{1}-\omega_{2}^{2}\eta_{22}\;\right)Y_{2}-2\omega_{2}\eta_{22}D_{1}^{}Y_{2}+c_{2}\left(a_{1}^{}+a_{2}^{}\right){\bar{Y}}_{1}e^{i\sigma_{0}T_{1}}\right]e^{i\omega_{2}T_{0}}+\mathrm{cc}+NST. \end{array}$$

where *NST* stands for terms that do not produce secular terms. The corresponding solvability condition can be obtained by eliminating secular terms.

(49) $$\begin{array}{l} 2\omega_{1}\eta_{21}D_{1}^{}Y_{1}+\alpha \omega_{1}\left(\omega_{1}^{2}\eta_{21}\;-c_{1}\right)Y_{1}=c_{2}\left(a_{1}^{}+a_{2}^{}\right){\bar{Y}}_{2}e^{i\sigma_{0}T_{1}};\\ 2\omega_{2}\eta_{22}D_{1}^{}Y_{2}+\alpha \omega_{2}\left(\omega_{2}^{2}\eta_{22}-c_{1}\right)Y_{2}=c_{2}\left(a_{1}^{}+a_{2}^{}\right){\bar{Y}}_{1}e^{i\sigma_{0}T_{1}}. \end{array}$$

Its solution is in the form of

(50) $$Y_{1}\left(T_{1}\right)=A_{1}e^{\left(r+is+0.5i\sigma_{0}\right)T_{1}};\;Y_{2}\left(T_{1}\right)=A_{2}e^{\left(r-is+0.5i\sigma_{0}\right)T_{1}},$$

where *r* is the real characteristic value.

Putting Equation (50) into Equation (49), and separating real and imaginary parts yield

(51a) $$2\omega_{1}\eta_{21}\left(r+is+0.5i\sigma_{0}\right)A_{1}+\alpha \omega_{1}\left(\omega_{1}^{2}\eta_{21}\;-c_{1}\right)A_{1}=c_{2}\left(a_{1}^{}+a_{2}^{}\right){\bar{A}}_{2};$$

(51b) $$2\omega_{2}\eta_{22}\left(r-is+0.5i\sigma_{0}\right)A_{2}+\alpha \omega_{2}\left(\omega_{2}^{2}\eta_{22}-c_{1}\right)A_{2}=c_{2}\left(a_{1}^{}+a_{2}^{}\right){\bar{A}}_{1},\;\mathrm{or}\;\mathrm{alternatively},\;2\omega_{2}\eta_{22}\left(r+is-0.5i\sigma_{0}\right){\bar{A}}_{2}+\alpha \omega_{2}\left(\omega_{2}^{2}\eta_{22}-c_{1}\right){\bar{A}}_{2}=c_{2}\left(a_{1}^{}+a_{2}^{}\right)A_{1},$$

and further from Equation (51a,b),

(52) $$A_{1}:{\bar{A}}_{2}=\frac{c_{2}\left(a_{1}^{}+a_{2}^{}\right)}{2\omega_{1}\eta_{21}\left(r+is+0.5i\sigma_{0}\right)+\alpha \omega_{1}\left(\omega_{1}^{2}\eta_{21}\;-c_{1}\right)}=\frac{2\omega_{2}\eta_{22}\left(r+is-0.5i\sigma_{0}\right)+\alpha \omega_{2}\left(\omega_{2}^{2}\eta_{22}-c_{1}\right)}{c_{2}\left(a_{1}^{}+a_{2}^{}\right)}.$$

As we know, the system is stable if and only if all eigenvalues are negative real numbers [31]. Otherwise, the electromechanical device is unstable. The critical situation is that the characteristic value is equal to zero. Consequently, the boundary of the stable region satisfies

(53) $$\frac{c_{2}\left(a_{1}^{}+a_{2}^{}\right)}{\alpha \omega_{1}\left(\omega_{1}^{2}\eta_{21}\;-c_{1}\right)+i\omega_{1}\eta_{21}\left(2s+\sigma_{0}\right)}=\frac{\alpha \omega_{2}\left(\omega_{2}^{2}\eta_{22}-c_{1}\right)+i\omega_{2}\eta_{22}\left(2s-\sigma_{0}\right)}{c_{2}\left(a_{1}^{}+a_{2}^{}\right)}.$$

Separating real and imaginary parts of Equation (53) yields

(54a) $$\eta_{21}\left(\sigma_{0}+2s\right)\left(\omega_{2}^{2}\eta_{22}-c_{1}\right)=\eta_{22}\left(\sigma_{0}-2s\right)\left(\omega_{1}^{2}\eta_{21}\;-c_{1}\right);$$

(54b) $$\omega_{1}\omega_{2}\eta_{21}\eta_{22}\left(\sigma_{0}+2s\right)\left(\sigma_{0}-2s\right)+\;\alpha_{}^{2}\omega_{1}\omega_{2}\left(\omega_{1}^{2}\eta_{21}\;-c_{1}\right)\left(\omega_{2}^{2}\eta_{22}-c_{1}\right)-c_{2}^{2}{\left(a_{1}^{}+a_{2}^{}\right)}_{}^{2}=0.$$

From Equation (54a),

(55) $$s=\frac{\sigma_{0}\left[\eta_{22}\left(\omega_{1}^{2}\eta_{21}\;-c_{1}\right)-\eta_{21}\left(\omega_{2}^{2}\eta_{22}-c_{1}\right)\right]}{2\left[\eta_{22}\left(\omega_{1}^{2}\eta_{21}\;-c_{1}\right)+\eta_{21}\left(\omega_{2}^{2}\eta_{22}-c_{1}\right)\right]}.$$

The stability boundary can be simplified from Equations (54b) and (55).

(56) $$\left|\sigma_{0}\right|=\frac{\eta_{21}\left(\omega_{2}^{2}\eta_{22}-c_{1}\right)+\eta_{22}\left(\omega_{1}^{2}\eta_{21}\;-c_{1}\right)}{2\eta_{21}\eta_{22}}\sqrt{\frac{c_{2}^{2}{\left(a_{1}^{}+a_{2}^{}\right)}_{}^{2}}{\omega_{1}\omega_{2}\left(\omega_{2}^{2}\eta_{22}-c_{1}\right)\left(\omega_{1}^{2}\eta_{21}\;-c_{1}\right)}-\alpha_{}^{2}}.$$

In general, the combination resonance of summed type occurs when

(57) $$\beta >\beta_{0}=\frac{\sqrt{\omega_{1}\omega_{2}\left(\omega_{1}^{2}\eta_{21}\;-c_{1}\right)\left(\omega_{2}^{2}\eta_{22}-c_{1}\right)}\sqrt{\alpha_{}^{2}+{\left[\frac{2\eta_{21}\eta_{22}\sigma_{0}}{\eta_{21}\left(\omega_{2}^{2}\eta_{22}-c_{1}\right)+\eta_{22}\left(\omega_{1}^{2}\eta_{21}\;-c_{1}\right)}\right]}^{2}}}{\omega^{2}\left|\frac{c_{2}^{}\eta_{11}}{\eta_{21}\;\left(\omega_{1}^{2}-\omega^{2}\right)}+\frac{c_{2}^{}\eta_{12}}{\eta_{22}\;\left(\omega_{2}^{2}-\omega^{2}\right)}\right|},$$

which is obtained on the basis of Equation (43) and Equation (56).

The considered physical parameters are *ρ* = 1.1 Tesla, *μ* = 1.256 × 10^−6^ H∙m^−1^, *H* = 0.002m, *R* = 0.004 m, *L* = 0.48 m, *m*_b_ = 2.56 × 10^−2^ kg, *E* = 3.81 × 10^10^ Pa, *I* = 3.6 × 10^−13^ m^4^ [3]. The other parameters can be assumed as *m*_t_ = 5.12 × 10^−4^ kg, g = 9.8067 m/s^2^, *D* = 0.3385 m, *S* = 5.0265 × 10^−5^ m^2^, Λ = 1.7310 × 10^7^ Pa∙s, *C*_eq_ = 5.9531 × 10^−8^ F, *R*_eq_ = 7631732 Ω, and Θ = 1.1906 × 10^−5^ N∙V^−1^. Namely, the dimensionless values are *α* = 0.001, *β* = 0.001, *c*_0_ = 0.5084, *d* = 0.15, *h* = 0.004167, *η* = 0.02, *c* = *κ* = 0.000001, and *θ* = 0.0002. The first two natural frequencies are about 4.048 and 21.356, respectively. When the exciting frequency is close to 25.4, the summed harmonic resonance occurs.

**Figure 2:**
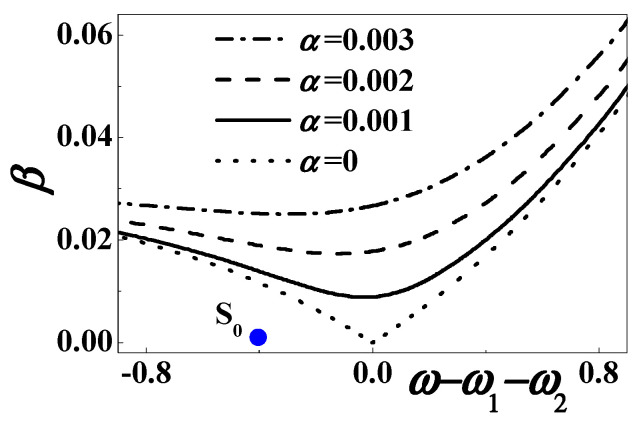
The stability boundary when *ω* ≈ *ω*_2_ + *ω*_1_.

Near the considered combination resonance, Figure 2 plots the stability boundaries (56) for different excitation and viscoelastic damping. The dotted, solid, dashed and dotted-dashed lines represent *α* = 0, *α* = 0.001, α = 0.002, and *α* = 0.003, respectively. The considered parameters are *c*_0_ = 0.5084, *d* = 0.15, *h* = 0.004167, *η* = 0.02, *c* = *κ* = 0.000001, and *θ* = 0.0002. It is revealed that the combination resonance might theoretically occur with a small damping, a large enough excitation amplitude, and a suitable excitation frequency close to the sum of the first two natural frequencies.

In contrast, the forced motion is usually stable when the excitation amplitude is small enough and the excitation frequency is off the combination resonance, such as the blue point S_0_ (depicted in Figure 2) where *β* = 0.001 and *σ*_0_ ≈ −0.4047 (*ω* = 25).

According to Equation (50), *Y*_1_ and *Y*_2_ decay with time in the stable, off-resonance region

(58) $$\left|\;\omega -\left(\omega_{1}+\omega_{2}\right)\right|>\frac{\eta_{21}\left(\omega_{2}^{2}\eta_{22}-c_{1}\right)+\eta_{22}\left(\omega_{1}^{2}\eta_{21}\;-c_{1}\right)}{2\eta_{21}\eta_{22}}\sqrt{\frac{c_{2}^{2}{\left(a_{1}^{}+a_{2}^{}\right)}_{}^{2}}{\omega_{1}\omega_{2}\left(\omega_{2}^{2}\eta_{22}-c_{1}\right)\left(\omega_{1}^{2}\eta_{21}\;-c_{1}\right)}-\alpha_{}^{2}}.$$

Therefore to the first-order approximation, the steady-state voltage output is approximately given by Equation (45).

(59) $$v\left(t\right)\approx \frac{\omega \left(a_{1}^{}\theta_{1}+a_{2}^{}\theta_{2}\right)}{\sqrt{\kappa^{2}+c^{2}\omega^{2}}}\sin\left(\omega t+\arctan\frac{\kappa }{c\omega }\right).$$

In addition, the differential harmonic oscillations are also deduced when *ω* ≈ *ω*_2_ − *ω*_1_. The multiscale analysis is quite similar to that of the summed harmonic oscillations. Nevertheless, the potential resonance cannot take place when the excitation frequency goes around the difference between the first two natural frequencies. The stable steady-state response possesses the same solution (59).

### 3.3. Numerical Simulations When ω = ω_2_ + ω_1_

For the sake of numerical computations, the applied two-term truncation system (34) is rewritten in the form of

(60) $$\begin{array}{l} {\dot{q}}_{n}=p_{n};\\ {\dot{p}}_{n}=\frac{\omega^{2}\beta \eta_{1n}}{\eta_{2n}\;}\sin\left(\omega t\right)+\frac{\theta_{n}}{\eta_{2n}\;}v-\omega_{n}^{2}q_{n}-\alpha \;\omega_{n}^{2}p_{n}+\frac{\alpha c_{1}}{\eta_{2n}\;}\left(p_{1}+p_{2}\right)-\frac{c_{2}}{\eta_{2n}\;}{\left(q_{1}+q_{2}\right)}^{2};\\ \dot{v}=\frac{\theta_{1}p_{1}+\theta_{2}p_{2}}{c}-\frac{\kappa }{c}v, \end{array}$$

where *p_n_* (*n* = 1, 2) are introduced and regarded as velocity.

Runge–Kutta method is one powerful numerical technique for solving differential equations [29]. The C programming language has been used to implement the numerical program of Equation (60). In the following, the potential steady-state responses are numerically computed by the use of the classical fourth-order Runge–Kutta method. The dimensionless time step is 2π/(10,000*ω*) in the C program source code. All the initial conditions are zero as the final behavior of the forced system is independent of them.

**Figure 3:**
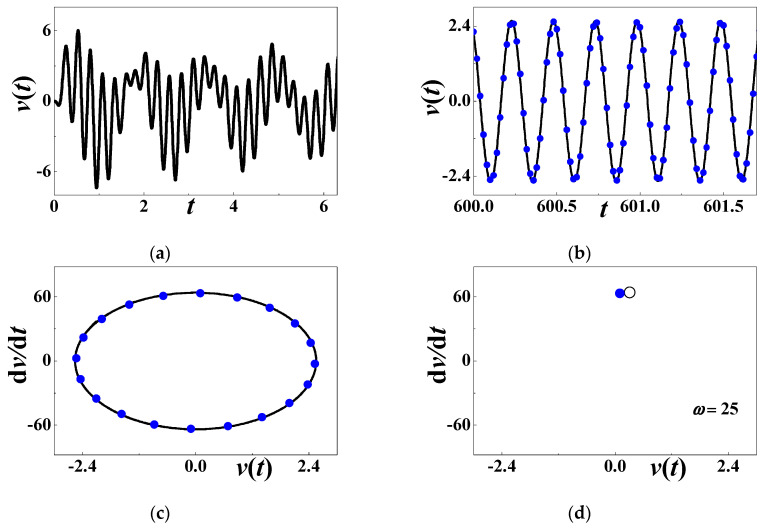
The periodic response when *ω* = 25: (**a**) initial history; (**b**) steady-state response; (**c**) phase diagram; (**d**) Poincare map. The solid blue dots stand for analytical prediction and others for numerical simulation.

**Figure 4:**
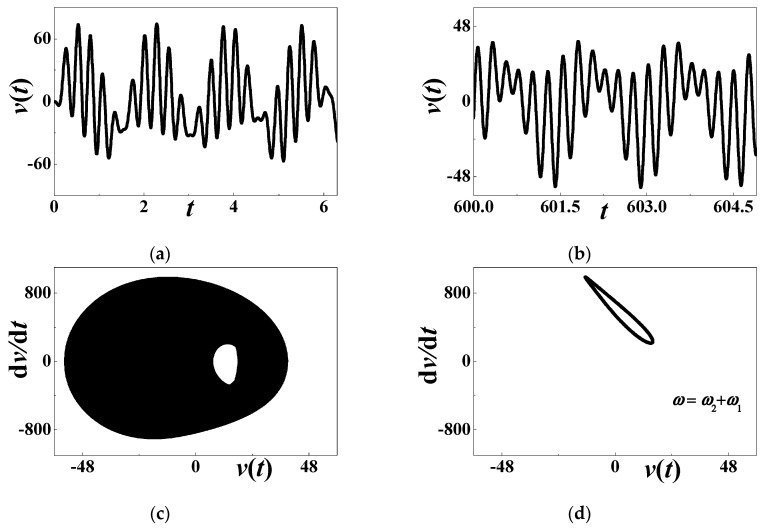
The quasiperiodic response when *ω* = *ω*_2_ + *ω*_1_: (**a**) initial history; (**b**) long-term response; (**c**) phase diagram; (**d**) Poincare map.

Figure 3 discusses the time history of voltage output at Point S_0_ in Figure 2. The initial history of output voltage is illustrated in Figure 3a where the considered parameters are *α* = 0.001, *β* = 0.001, *c*_0_ = 0.5084, *d* = 0.15, *h* = 0.004167, *η* = 0.02, *c* = *κ* = 0.000001, *θ* = 0.0002, and *ω* = 25 (*σ*_0_ ≈ −0.4047). The transient motion is short and the steady-state motion occurs in the terminal interval [600, 602]. Figure 3b compares the analytical voltage output (59) with the steady-state response (60) based on the Runge–Kutta method. Figure 3c compares the steady-state phase diagram from two different methods. The solid line stands for numerical results (60), and the solid blue dots for analytical approximation (59). These figures support that the steady-state response is periodic, although there is a quantitative difference between analytical prediction and numerical simulation. In Figure 4d, the Poincare map theory is also employed to verify the periodic motion. The multiscale analysis predicts that only a point emerges in the phase diagram. The solid blue dot is the analytical result. The hollowed dots are based on numerical computation. Figure 4d reveals that the steady-state voltage output is a periodic response whose period is the same as the external excitation.

Figure 4 focuses on the numerical quasiperiodic response at an unstable point. The considered combination of system parameters is *α* = 0.0001, *β* = 0.01, *c*_0_= 0.5084, *d* = 0.15, *h* = 0.004167, *η* = 0.02, *c* = *κ* = 0.000001, *θ* = 0.0002, and *ω* ≈ 25.4 (*σ*_0_ = 0). Different from previous results, the forced vibration is not periodic any more. After a short transition, the long-term voltage output is similar to the response history in the terminal interval [600, 605], as depicted in Figure 4b. The phase diagram in Figure 4c is not a simple closed curve, which supports the conclusion that the forced vibration is aperiodic. The Poincare map in Figure 4d illustrates the quasiperiodic response, as a closed curve appears in the phase diagram.

### 3.4. Numerical Simulations When ω = ω_2_ − ω_1_

To discuss the potential combination resonance of difference type, two numerical computations are carried out here. To numerically support the analytical prediction (59), the considered parameters are *c*_0_ = 0.5084, *d* = 0.15, *h* = 0.004167, *η* = 0.02, *c* = *κ* = 0.000001, *θ* = 0.0002, and *ω* = *ω*_2_ − *ω*_1_ ≈ 17.3. The following are other parameters in two different cases.

- Case 1: *α* = 0.001, and *β* = 0.001, which is the same with Figure 3.
- Case 2: *α* = 0.0001, and *β* = 0.01, which is the same with Figure 4.

**Figure 5:**
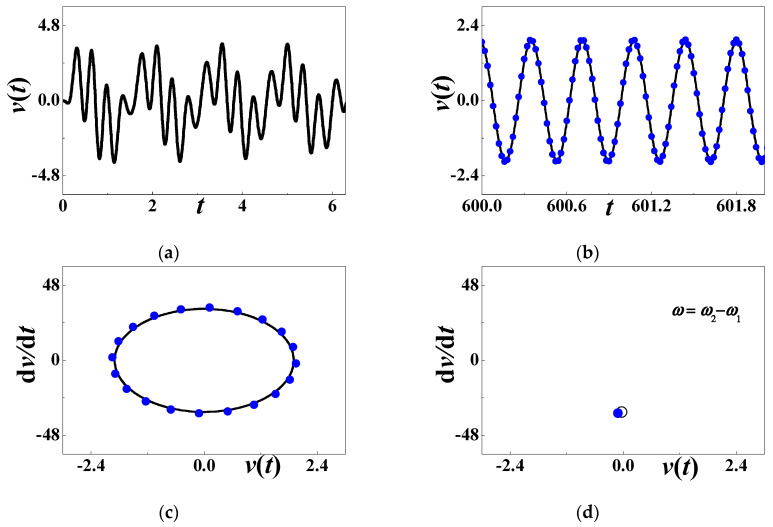
The small-amplitude periodic response when *ω* = *ω*_2_ − *ω*_1_: (**a**) initial history; (**b**) steady-state response; (**c**) phase diagram; (**d**) Poincare map. The solid blue dots stand for analytical prediction and others for numerical simulation.

**Figure 6:**
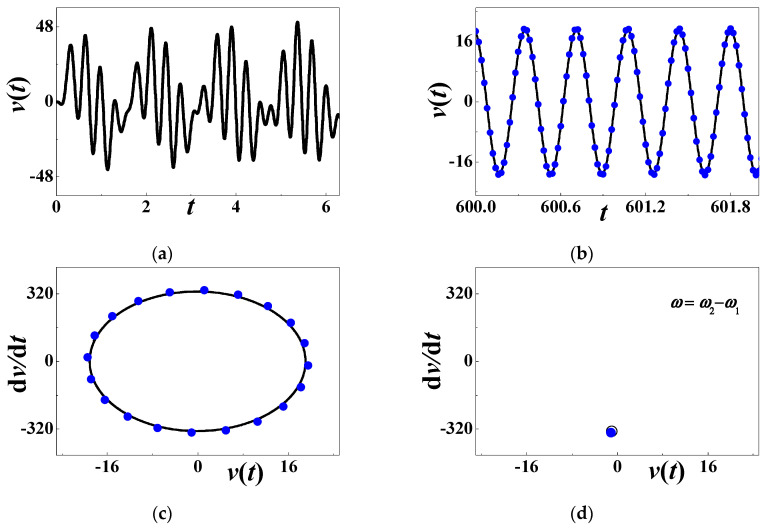
The large-amplitude periodic response when *ω* = *ω*_2_ − *ω*_1_: (**a**) initial history; (**b**) steady-state response; (**c**) phase diagram; (**d**) Poincare map. The solid blue dots stand for analytical prediction and others for numerical simulation.

Figure 5 and Figure 6 describe the dynamic characteristics of Case 1 and Case 2, respectively. Based on the two-term Galerkin truncation (60), two methods predict the same tendencies very well. The analytical periodic motion is numerically verified on the basis of Figure 5b–d and Figure 6b–d. In these figures, the solid blue dots denote the analytical time histories, the phase diagrams, or Poincare maps during the steady-state response. The other curves and hollowed dots represent the numerical results.

It can be observed that the response remains stable in Figure 6 with a large excitation amplitude and small damping. The external excitation determines the period of the steady-state response. The numerical result quantitatively explains the theoretical prediction (59). It is very evident that the combination resonance of difference type does not take place in the absence of internal resonance.

Besides, the stable voltage output is independent of viscoelastic damping according to Equations (43) and (59). It can be theoretically concluded that the damping has no effect on the stable steady-state response. To some extent, the numerical computations with different damping almost result in an identical outcome.

## 4. Subharmonic Resonances

### 4.1. The First Subharmonic Resonance

In this section, introduce the detuning parameter according to

(61) $$\varepsilon \sigma_{1}=\;\omega -2\omega_{1}\;,$$

and then

(62) $$\left(\omega -\omega_{1}\right)T_{0}=\left(\omega_{1}+\varepsilon \sigma_{1}\right)t=\omega_{1}T_{0}+\sigma_{1}T_{1}.$$

Equation (40b) becomes

(63) $$\begin{array}{c} \left(D_{0}^{2}Q_{12}+\omega_{1}^{2}Q_{12}\right)\eta_{21}\;=\;i\left[\alpha \omega_{1}\left(c_{1}-\;\omega_{1}^{2}\eta_{21}\right)Y_{1}-2\omega_{1}\eta_{21}D_{1}^{}Y_{1}+c_{2}\left(a_{1}^{}+a_{2}^{}\right){\bar{Y}}_{1}e^{i\sigma_{1}T_{1}}\right]e^{i\omega_{1}T_{0}}+\mathrm{cc}+NST;\\ \left(D_{0}^{2}Q_{22}+\omega_{2}^{2}Q_{22}\right)\eta_{22}\;=i\alpha \omega_{2}\left(c_{1}-\omega_{2}^{2}\eta_{22}\;\right)Y_{2}e^{i\omega_{2}T_{0}}-2i\omega_{2}\eta_{22}D_{1}^{}Y_{2}e^{i\omega_{2}T_{0}}+\mathrm{cc}+NST. \end{array}$$

Eliminating those terms that produce secular terms has

(64a) $$\;\omega_{1}\left[2\eta_{21}D_{1}^{}Y_{1}+\alpha \left(\omega_{1}^{2}\eta_{21}\;-c_{1}\right)Y_{1}\right]=c_{2}\left(a_{1}^{}+a_{2}^{}\right){\bar{Y}}_{1}e^{i\sigma_{1}T_{1}};$$

(64b) $$2\eta_{22}D_{1}^{}Y_{2}+\alpha \left(\omega_{2}^{2}\eta_{22}-c_{1}\right)Y_{2}=0.$$

The solution to Equation (64b) is

(65) $$Y_{2}^{}\left(T_{1}\right)=A_{2}\exp\left[-\frac{\alpha \left(\omega_{2}^{2}\eta_{22}-c_{1}\right)}{2\eta_{22}}T_{1}\right]\to 0\;\left(t\to \infty \right).$$

The solution to Equation (64a) is in the form of

(66) $$Y_{1}\left(T_{1}\right)=\left(A_{1r}+iA_{1i}\right)e^{\left(r+0.5i\sigma_{1}\right)T_{1}},$$

where *r*, *A*_1r_, and *A*_1i_ are real numbers. Equation (64a) can be rewritten as

(67) $$\begin{array}{l} 2r\omega_{1}\eta_{21}A_{1r}+\alpha \omega_{1}\left(\omega_{1}^{2}\eta_{21}\;-c_{1}\right)A_{1r}-c_{2}\left(a_{1}^{}+a_{2}^{}\right)A_{1r}-\sigma_{1}\omega_{1}\eta_{21}A_{1i}=0;\\ 2r\omega_{1}\eta_{21}A_{1i}+\alpha \omega_{1}\left(\omega_{1}^{2}\eta_{21}\;-c_{1}\right)A_{1i}+c_{2}\left(a_{1}^{}+a_{2}^{}\right)A_{1i}+\sigma_{1}\omega_{1}\eta_{21}A_{1r}=0. \end{array}$$

For a nontrivial solution, two characteristic values are

(68) $$r_{1,2}=-\frac{\alpha \left(\omega_{1}^{2}\eta_{21}-c_{1}\;\right)}{2\eta_{21}}\pm \frac{1}{2}\sqrt{\frac{c_{2}^{2}{\left(a_{1}^{}+a_{2}^{}\right)}_{}^{2}}{\omega_{1}^{2}\eta_{21}^{2}}-\sigma_{1}^{2}}.$$

Here, *Y*_1_ oscillates and decays if

(69) $$\;\sigma_{1}^{2}\omega_{1}^{2}\eta_{21}^{2}>c_{2}^{2}{\left(a_{1}^{}+a_{2}^{}\right)}_{}^{2}\;\Rightarrow \;Y_{1}\left(T_{1}\right)\to 0\;\left(t\to +\infty \right)\;.$$

However, *Y*_1_ decays without oscillating if

(70) $$c_{2}^{2}{\left(a_{1}^{}+a_{2}^{}\right)}_{}^{2}-\;\alpha_{}^{2}\omega_{1}^{2}{\left(\omega_{1}^{2}\eta_{21}\;-c_{1}\right)}_{}^{2}<\sigma_{1}^{2}\omega_{1}^{2}\eta_{21}^{2}<c_{2}^{2}{\left(a_{1}^{}+a_{2}^{}\right)}_{}^{2}.$$

Therefore to the first-order approximation, the steady-state voltage output is approximately given by

(71) $$v\left(t\right)\approx \frac{\omega \left(a_{1}^{}\theta_{1}+a_{2}^{}\theta_{2}\right)}{\sqrt{\kappa^{2}+c^{2}\omega^{2}}}\sin\left(\omega t+\arctan\frac{\kappa }{c\omega }\right)\;\mathrm{if}\;c_{2}^{2}{\left(a_{1}^{}+a_{2}^{}\right)}_{}^{2}<\alpha_{}^{2}\omega_{1}^{2}{\left(c_{1}-\eta_{21}\omega_{1}^{2}\;\right)}_{}^{2}+\sigma_{1}^{2}\omega_{1}^{2}\eta_{21}^{2}.$$

Otherwise the forced motion is unstable. The stability boundary is

(72) $$\sigma_{1}^{2}\omega_{1}^{2}\eta_{21}^{2}=c_{2}^{2}{\left(a_{1}^{}+a_{2}^{}\right)}_{}^{2}-\;\alpha_{}^{2}\omega_{1}^{2}{\left(\omega_{1}^{2}\eta_{21}\;-c_{1}\right)}_{}^{2}.$$

**Figure 7:**
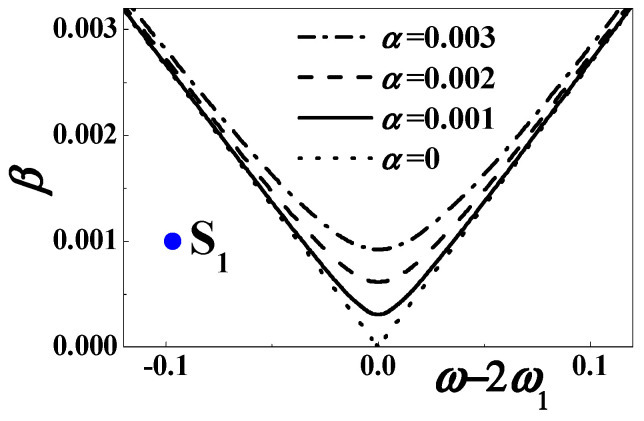
The stability boundary when *ω* ≈ 2*ω*_1_.

Figure 7 plots the stability boundaries (72) for different damping and excitation amplitude. The dotted, solid, dashed and dotted-dashed lines represent *α* = 0, *α* = 0.001, *α* = 0.002, and *α* = 0.003. The other parameters are *c*_0_ = 0.5084, *d* = 0.15, *h* = 0.004167, *η* = 0.02, *c* = *κ* = 0.000001, and *θ* = 0.0002. When the exciting frequency is close to twice the first natural frequency, the stability strongly depends on the viscosity damping. If the damping coefficient is very small, the output voltage might grow without bound according to Equation (66). In addition, when the excitation amplitude is small enough, the forced motion is stable off the first subharmonic resonance, such as the blue point S_1_ (depicted in Figure 7) where *β* = 0.001 and *σ*_1_ ≈ −0.09674 (*ω* = 8).

### 4.2. Numerical Simulations When ω = 2ω_1_

At the stable point, Point S_1_ in Figure 7, the numerical simulation (60) is carried out and compared with the analytical steady-state voltage output (71).

**Figure 8:**
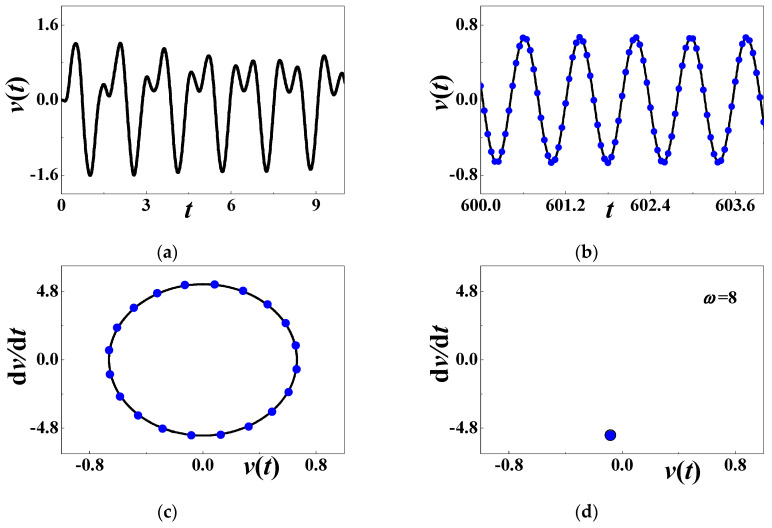
The periodic response when *ω* = 8: (**a**) initial history; (**b**) steady-state response; (**c**) phase diagram; (**d**) Poincare map. The solid blue dots stand for analytical prediction and others for numerical simulation.

Figure 8a is the initial history of output voltage with *α* = 0.001, *β* = 0.001, *c*_0_ = 0.5084, *d* = 0.15, *h* = 0.004167, *η* = 0.02, *c* = *κ* = 0.000001, *θ* = 0.0002, and *ω* = 8 (*σ*_1_ ≈ −0.09674). In the terminal interval [600, 604], the steady-state response, the phase diagram, and the Poincare map are respectively compared in Figure 8b–d. The solid black lines stand for numerical results (60), and the solid blue dots for analytical prediction (71). In Figure 8d, the hollowed dots are numerically obtained by Poincare mapping. Two different methods almost yield the same mapping results in Figure 8d. Obviously, the system generates a steady-state voltage in the analytically stable region, and the oscillating frequency is the same as the excitation frequency.

**Figure 9:**
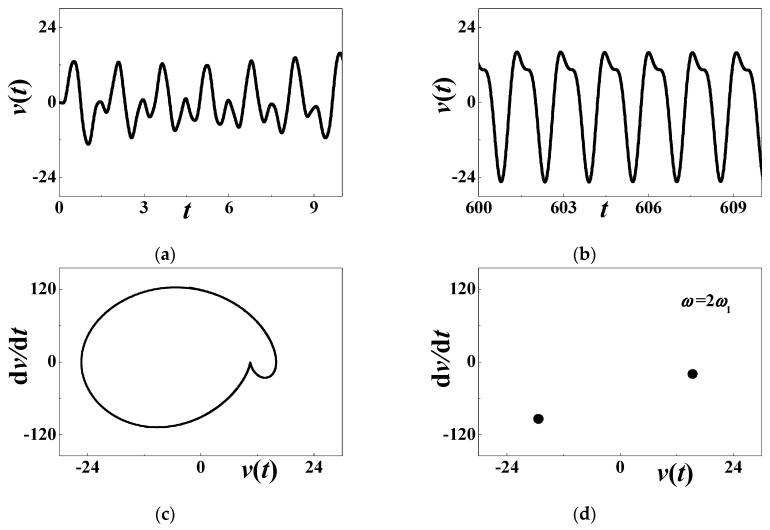
The period-doubling motion when *ω* = 2*ω*_1_: (**a**) initial history; (**b**) steady-state response; (**c**) phase diagram; (**d**) Poincare map.

In the unstable region, the excitation amplitude is required to be particularly large. Figure 9 numerically displays the response (60) with *α* = 0.0001, *β* = 0.01, *c*_0_ = 0.5084, *d* = 0.15, *h* = 0.004167, *η* = 0.02, *c* = *κ* = 0.000001, *θ* = 0.0002, and *ω* = 2*ω*_1_ ≈ 8.09674 (*σ*_1_ = 0). Figure 9a describes an irregular initial motion, and Figure 9b illustrates a steady-state voltage output, which is also observed from the phase diagram in Figure 9c and the Poincare map in Figure 9d. According to the Poincare map theory, the steady-state response is period-doubling because there are two points in the phase plane.

### 4.3. The Second Subharmonic Resonance

In this case, introduce the detuning parameter according to

(73) $$\varepsilon \sigma_{2}=\;\omega -2\omega_{2}\;,$$

and then Equation (40b) becomes

(74) $$\begin{array}{c} \left(D_{0}^{2}Q_{12}+\omega_{1}^{2}Q_{12}\right)\eta_{21}\;=\;i\alpha \omega_{1}\left(c_{1}-\;\omega_{1}^{2}\eta_{21}\right)Y_{1}e^{i\omega_{1}T_{0}}-2i\omega_{1}\eta_{21}D_{1}^{}Y_{1}e^{i\omega_{1}T_{0}}+\mathrm{cc}+NST;\\ \left(D_{0}^{2}Q_{22}+\omega_{2}^{2}Q_{22}\right)\eta_{22}\;=i\left[\alpha \omega_{2}\left(c_{1}-\omega_{2}^{2}\eta_{22}\;\right)Y_{2}-2\omega_{2}\eta_{22}D_{1}^{}Y_{2}+c_{2}\left(a_{1}^{}+a_{2}^{}\right){\bar{Y}}_{1}e^{i\sigma_{2}T_{1}}\right]e^{i\omega_{2}T_{0}}+\mathrm{cc}+NST. \end{array}$$

Eliminating those terms that produce secular terms gives

(75a) $$2\eta_{21}D_{1}^{}Y_{1}+\alpha \left(\omega_{1}^{2}\eta_{21}\;-c_{1}\right)Y_{1}=0;$$

(75b) $$\;\omega_{2}\left[2\eta_{22}D_{1}^{}Y_{2}+\alpha \left(\omega_{2}^{2}\eta_{22}-c_{1}\right)Y_{2}\right]=c_{2}\left(a_{1}^{}+a_{2}^{}\right){\bar{Y}}_{2}e^{i\sigma_{2}T_{1}}.$$

The solution to Equation (75a) is

(76) $$Y_{1}^{}\left(T_{1}\right)=A_{1}\exp\left[-\frac{\alpha \left(\omega_{1}^{2}\eta_{21}-c_{1}\right)}{2\eta_{21}}T_{1}\right]\to 0\;\left(t\to \infty \right).$$

The solution to Equation (75b) can be expressed in the form of

(77) $$Y_{2}\left(T_{1}\right)=\left(A_{2r}+iA_{2i}\right)e^{\left(r+0.5i\sigma_{2}\right)T_{1}},$$

where *r*, *A*_2r_, and *A*_2i_ are real numbers. Equation (75b) can be rewritten as

(78) $$\begin{array}{l} 2r\omega_{2}\eta_{22}A_{2r}+\alpha \omega_{2}\left(\omega_{2}^{2}\eta_{22}-c_{1}\right)A_{2r}-c_{2}\left(a_{1}^{}+a_{2}^{}\right)A_{2r}-\sigma_{2}\omega_{2}\eta_{22}A_{2i}=0;\\ 2r\omega_{2}\eta_{22}A_{2i}+\alpha \omega_{2}\left(\omega_{2}^{2}\eta_{22}-c_{1}\right)A_{2i}+c_{2}\left(a_{1}^{}+a_{2}^{}\right)A_{2i}+\sigma_{2}\omega_{2}\eta_{22}A_{2r}=0. \end{array}$$

For a nontrivial solution, two characteristic values are

(79) $$r_{1,2}=-\frac{\alpha \left(\omega_{2}^{2}\eta_{22}-c_{1}\right)}{2\eta_{22}}\pm \frac{1}{2}\sqrt{\frac{c_{2}^{2}{\left(a_{1}^{}+a_{2}^{}\right)}_{}^{2}}{\omega_{2}^{2}\eta_{22}^{2}}-\sigma_{2}^{2}}.$$

The electromechanical system is stable if and only if all roots (79) of the characteristic equation possess a negative real part. Then, the potential steady-state motion is

(80) $$v\left(t\right)\approx \frac{\omega \left(a_{1}^{}\theta_{1}+a_{2}^{}\theta_{2}\right)}{\sqrt{\kappa^{2}+c^{2}\omega^{2}}}\sin\left(\omega t+\arctan\frac{\kappa }{c\omega }\right)\;\mathrm{if}\;c_{2}^{2}{\left(a_{1}^{}+a_{2}^{}\right)}_{}^{2}<\alpha_{}^{2}\omega_{2}^{2}{\left(\omega_{2}^{2}\eta_{22}\;-c_{1}\right)}_{}^{2}+\sigma_{2}^{2}\omega_{2}^{2}\eta_{22}^{2}.$$

Here, the stability boundary is

(81) $$\sigma_{2}^{2}\omega_{2}^{2}\eta_{22}^{2}=c_{2}^{2}{\left(a_{1}^{}+a_{2}^{}\right)}_{}^{2}-\;\alpha_{}^{2}\omega_{2}^{2}{\left(\omega_{2}^{2}\eta_{22}\;-c_{1}\right)}_{}^{2}.$$

**Figure 10:**
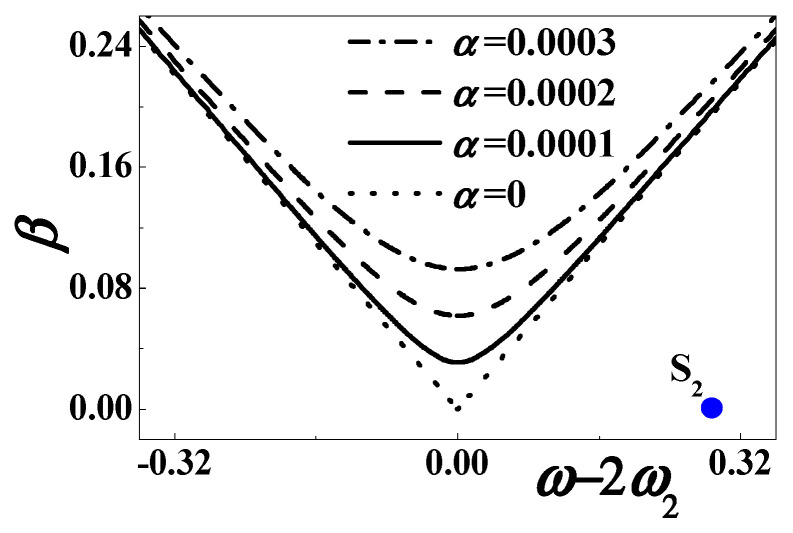
The stability boundary when *ω* ≈ 2*ω*_2_.

Near the second subharmonic resonance, Figure 10 plots the stability boundaries (81) for different combinations of external excitation and viscoelastic damping. The dotted, solid, dashed and dotted-dashed lines represent *α* = 0, *α* = 0.0001, *α* = 0.0002, and *α* = 0.0003, respectively. The considered parameters are *c*_0_ = 0.5084, *d* = 0.15, *h* = 0.004167, *η* = 0.02, *c* = *κ* = 0.000001, and *θ* = 0.0002. It is revealed that the output voltage might grow without bound according to Equation (77). The necessary conditions are theoretically an extremely small damping, a large enough excitation amplitude, and the suitable excitation frequency close to twice the second natural frequency. As the method of multiple time scales requires that the response amplitude is small, the forced motion is usually stable off the subharmonic resonance, such as the blue point S_2_ (depicted in Figure 10) where *β* = 0.001 and *σ*_2_ ≈ 0.2874 (*ω* = 43).

### 4.4. Numerical Simulations When ω = 2ω_2_

**Figure 11:**
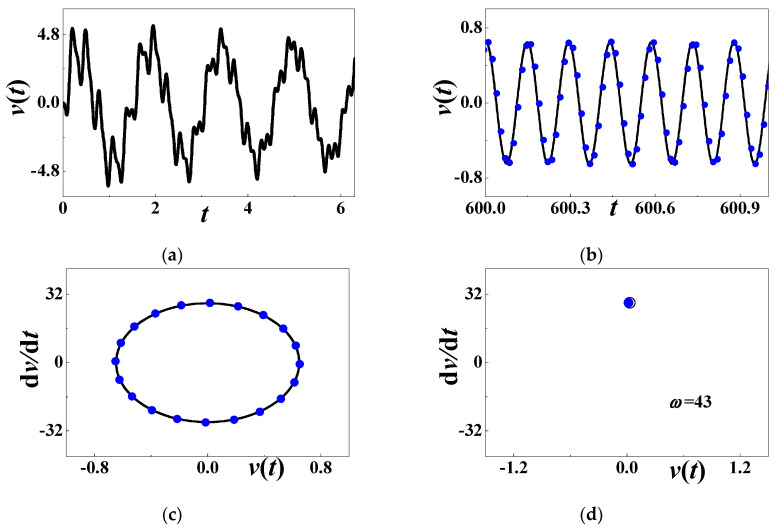
The periodic response when *ω* = 43: (**a**) initial history; (**b**) steady-state response; (**c**) phase diagram; (**d**) Poincare map. The solid blue dots stand for analytical prediction and others for numerical simulation.

At the stable point S_2_ in Figure 10, the numerical simulation is used to verify the analytical steady-state voltage output (80). Figure 11a is the initial history of output voltage with *α* = 0.001, *β* = 0.001, *c*_0_ = 0.5084, *d* = 0.15, *h* = 0.004167, *η* = 0.02, *c* = *κ* = 0.000001, *θ* = 0.0002, and *ω* = 43 (*σ*_2_ ≈ 0.2874). The period is much smaller than those of previous examples if a periodic response exists. In the terminal interval [600, 601], the steady-state response, the phase diagram and the Poincare map are respectively compared in Figure 11b–d. The solid black lines stand for numerical results (60), and the solid blue dots for analytical prediction (80). In Figure 11d, the hollowed dots are numerically obtained by Poincare mapping, and approach the analytical result. In the stable region for the second subharmonic resonance, the system might generate a steady-state voltage output, and the oscillating frequency is the same as the excitation frequency.

**Figure 12:**
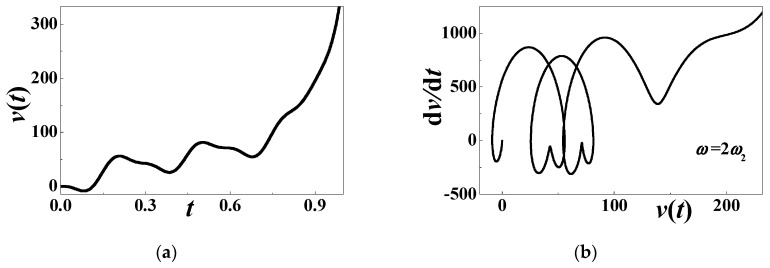
The growing response without bound when *ω* = 2*ω*_2_: (**a**) initial history; (**b**) phase diagram.

It is numerically convinced that the second subharmonic resonance exists when *ω* = 2*ω*_2_ ≈ 42.7 (*σ*_2_ = 0). Figure 12 gives a growing response without bound. During the unstable motion, the considered parameters are *α* = 0.0001, *β* = 0.01, *c*_0_ = 0.508394, *d* = 0.15, *h* = 0.004167, *η* = 0.02, *c* = *κ* = 0.000001, and *θ* = 0.0002. The initial history and its phase diagram are plotted in Figure 12a,b, respectively. It can be seen that the output voltage is far away from the original point. This conclusion is very useful to capture vibratory energy. However, the large-amplitude swaying motion is capable of causing structural damage. A combination of security and performance should be considered in detail for real use.

## 5. Discussions

### 5.1. The Experimental Explanation

So far, we have not carried out the resonances experimentally. Nevertheless, Lin et al. tested a nonlinear energy harvester around a subharmonic resonance [11,17]. This kind of secondary resonance does not exist in any other linear system.

In [11], a piezoelectric cantilever with magnetic coupling is used to enhance the width of the frequency band. Their nonlinear system is simplified as a single-degree-of-freedom model. The natural frequency is approximately 10 Hz and the resonant frequency is a bit larger. When the excitation frequency is about 24 Hz, an obvious peak appeared in Figure 2 of [11]. The vibration amplification phenomenon means at least two conclusions: (a) A potential subharmonic resonance exists in the piezoelectric energy harvester; (b) the magnetic force can generate quadratic nonlinearity. However, the subharmonic resonance did not catch the authors’ attention at once. They only discussed the output voltage when the excitation frequency is 6.5 Hz, 10 Hz, and 13 Hz. Further study is carried out in [17].

An improved experiment is set up by adjusting the distance between two magnets [17]. More time histories of the output voltage are observed and compared. At the excitation frequency of 20 Hz, the output voltage is periodic. The nonlinear cantilever shows the response amplitude five times as large as that of a linear cantilever without magnetic coupling. When the excitation frequency is between 23 Hz and 30 Hz, the amplitude of output voltage is almost the same as the linear cantilevered system. These experiments have indicated that magnetic nonlinearity is capable of improving the performance of energy harvesting. Compared with a linear harvester, the nonlinear system can indeed provide more electrical energy because of the subharmonic resonances or other nonlinear characteristics.

As theoretical compensation, we intend to apply our theory to the experiment in [17]. Although the steady-state response of voltage is periodic, its period is not that of the external excitation any longer. Instead, the period of the voltage output is exactly twice the period of the excitation at any subharmonic resonance. The period-doubling motion can also easily be identified from Figure 3 of [17].

Moreover, not all the voltage output is period-doubling. According to our theoretical results, the steady-state response might be a periodic motion of single frequency. The more complex response might occur at the combination resonance of the summed type. In the absence of internal resonances, there is no amplitude amplification phenomenon when the excitation frequency approaches the difference between two natural frequencies. These theoretical analyses not only forecast the stability boundary, but also demonstrate different types of the output voltage. Obviously, the stability boundary is quite difficult to be analyzed by experiments. The theoretical significance is that it can be used to more reasonably design energy harvesters.

### 5.2. The Effects of Material Damping

Piezoelectric ceramics are ferroelectric synthetic materials. In general, it can be manufactured from Pb (lead), Zr (zirconium), and Ti (titanium). This compound class shows higher piezo-mechanical efficiency than quartz. Meanwhile, a different combination of Pb, Zr, and Ti is capable of producing diversity in the damping of piezoelectric materials. Table 2 displays the different mechanical quality factors and dielectric losses for the piezo-materials manufactured by APC International, Ltd., formerly American Piezo Ceramics, Inc., Mackeyville, PA, USA [33].

In practice, piezoelectric materials also show changing parameter values owing to their thickness, actual shape, surface finish, shaping process, and postprocessing. In general, a hard piezo-material, which is harder to depolarize than a soft piezo-material, exhibits higher mechanical quality factors and lower dielectric losses. This combination of piezoelectric and mechanical properties makes hard piezo-materials ideal for piezoelectric energy harvesting. In order to design an energy harvester working at the subharmonic or combination resonance, APC 841, APC 844, APC 880, and APC 881 are preferable.

As a matter of fact, the improved damping materials are not effective sometimes. Equations (59), (71), and (80) are all independent of the damping coefficient. The response amplitude of these stable steady-state voltage does not increase with a smaller damping coefficient. However, the selected piezoelectric materials are still helpful. It contributes to design the energy harvesters working at the subharmonic or combination resonance. A small deviation off resonance can cause a great drop in performance output.

Here is what we have to say. The conclusions in [17] are mainly based on an experimental test. The saying, “the stochastic, subharmonic, and ultraharmonic responses produce an average of threefold to fivefold increase in voltage production”, is not supported by our in-depth analyses. The comparison should be on the basis of the stability theory proposed in this article. For example, the nonlinear energy harvester has no superiority if the nonlinearity is weak enough, the excitation amplitude is small enough, or the external excitation is off-resonance.

### 5.3. The Effects of Excitation Amplitude

The excitation amplitude is quite important in the stability analyses. The stability of the output voltage is directly affected by the amplitude of the external excitation. As is described in Section 3.1, these secondary resonances occur when the excitation amplitude is large enough.

In the stable region, the output voltage is periodic and the period of the steady-state response is exactly the same as that of the external excitation. From Equation (43), the response amplitude of output voltage is almost proportional to the amplitude of the external excitation. Within the proportional limit, the response amplitude linearly grows with the increased excitation amplitude. When the excitation amplitude is large enough, a secondary resonance might occur.

In the unstable region, an increased excitation amplitude can cause a great rise in the width of the frequency band. This phenomenon is just desirable, as depicted in Figure 2, Figure 7, and Figure 10. As we all know, the linear energy harvester only works at a single frequency. According to the stability analyses, the nonlinear energy harvester can greatly improve the range of working frequency. The enhanced performance enables the nonlinear device to work in a random environment.

Besides, we disagree that the output voltage has the probability of growing without bound, even though the theoretical analysis forecasts the growing response and the numerical simulation follows. The conception is based on the following two reasons at least: (a) The response amplitude of the piezoelectric cantilever must be less than its length; (b) The methodology requires a weak response, which is unsuitable for the large-amplitude motion.

### 5.4. The Effects of Maximal Voltage

In addition, there is another reason why the output voltage must be limited. Piezoelectric ceramic materials have the ability to both generate and respond to voltages. The output voltage controls the applied electric field. An ultra-high electric field can change the piezoelectric properties of piezoelectric ceramics after polarization. Accordingly, the piezoelectric energy harvester should be operated in safe working environments.

The alternating current field is illustrated in this article. The applied maximal voltage (per unit thickness) of some APC materials is provided for reference by APC International, Ltd.

5-7 VAC/mil for APC 850, APC 851, APC 854, APC 855 where one inch = 1000 mil.

9-11 VAC/mil for APC 840, APC 841, APC 842, APC 844, APC 880, APC 881 [33].

From a physical point of view, the mechanical properties of piezoelectric materials are also linked to the time duration of the applied electric field. Ultrahigh electric fields are often easier to cause changes in system parameters, enhanced nonlinear responses, material fatigue damages, and even structural fracture behaviors.

All in all, the theoretical analyses provide a novel solution to enhance electrical performance as well as a wider range of working frequencies to some extent. However, the output voltage or excitation amplitude must be less than its maximum permitted. The efficiency to harvest mechanical energy is worthy of being experimentally studying. A combination of security and performance should be considered in detail for real use.

## 6. Conclusions

For the first time, the summed combination resonance is developed to harvest vibratory energy. A cantilever-type piezoelectric energy harvester with tip magnets is investigated. The mathematical model is an integro-partial differential equation with time-dependent boundary conditions. A newly-built methodology is proposed to deal with the issue of continuum mechanics. Several dynamic phenomena are found to be of interest and novelty, such as the quasiperiodic motion and the theoretically growing response without bound.

The subharmonic resonances are also approximately analyzed by the improved Galerkin method. Based on the two-term truncation system with quadratic nonlinearity, the multiscale method is developed to solve these secondary resonances. The numerical simulation obtained by Runge–Kutta method well matches those stable results obtained analytically. The stability theory is quite useful to design a piezoelectric energy harvester. To some extent, these secondary resonances may provide both a much larger voltage output and a wider bandwidth of working frequency, as partially experimented in [11,17].

The differential combination resonance is discussed analytically and numerically, too. The main conclusions are as follows.

- When the excitation frequency approaches the sum of two natural frequencies, the combination resonance of the summed type exists. The stability boundary is provided approximately but analytically and sensitive to the viscoelastic damping. In the stable region, the one-order approximation of steady-state response is formulated. In the unstable region, the quasiperiodic motion may occur. The unstable response is readily found with small damping and a large enough excitation amplitude.
- When the excitation frequency approaches the difference between two natural frequencies, the differential harmonic oscillation does not exist. Their long-term response is simply harmonic and their frequency output is the same as the excitation frequency. To some extent, viscoelastic damping has no effect on the steady-state response. This outcome is drawn in the absence of internal resonance.
- When the excitation frequency approaches twice the natural frequency, the subharmonic resonance exists. The stability boundaries are provided for the first two vibration modes. Increasing damping decreases the unstable region, where there might be a period-doubling motion, or a theoretically growing response without bound. On the contrary, simply-harmonic voltage output is produced off the subharmonic resonances. The steady-state response in the stable region is explicitly expressed and numerically verified.

In summary, the summed combination resonances and the subharmonic resonances can be exploited to harvest more mechanical energy. Different from the primary resonance, these secondary resonances might improve the bandwidth towards two different directions. In general, the primary resonance merely enhances the bandwidth towards one direction, which is well known as the soft-spring or hard-spring behavior, respectively. The proposed stability analyses can be used to design the piezoelectric energy harvester. The stability boundary is very sensitive to material damping. In the stable region, the nonlinear energy harvester cannot produce more voltage output than the linear device, which is independent of damping. On the contrary, the secondary resonance energy harvester must work in an unstable region. With the help of further experiments, the low-cost piezoelectric energy harvester can support a wide range of industries, including structural health monitoring sensors and wireless networks. To this end, APC 841, APC 844, APC 880, and APC 881 are preferable piezoelectric ceramic materials to manufacture these electronic applications.

## Figures and Tables

**Table 1:** The structural parameters considered in this paper and [3].

Symbols	Descriptions	Values	Units
*ρ*	Residual flux density	1.1	T
*μ*	Permeability of air	1.256 × 10^−6^	H·m^−1^
*H*	Length of the cylindrical magnet	2	mm
*R*	Radius of the cylindrical magnet	4	mm
*L*	Length of the cantilever	480	mm
*m*_b_	Mass of the cantilever	25.6	g
*E*	Young’s modulus	3.81 × 10^10^	Pa
*I*	Moment of inertia	0.36	mm^4^

**Table 2:** Physical properties of APC materials [33].

APC Materials	Mechanical Quality Factors	Dielectric Dissipation Factors
840	500	0.6%
841	1400	0.4%
842	600	0.45%
844	1500	0.4%
850/851	80	<2%
854	70	<2%
855	65	<2.5%
860	50	<2%
880/881	1000	0.4%
